# Betaine anhydrous in homocystinuria: results from the RoCH registry

**DOI:** 10.1186/s13023-019-1036-2

**Published:** 2019-03-14

**Authors:** Vassili Valayannopoulos, Manuel Schiff, Nathalie Guffon, Yann Nadjar, Angels García-Cazorla, Mercedes Martinez-Pardo Casanova, Aline Cano, Maria L. Couce, Jaime Dalmau, Luis Peña-Quintana, Vincent Rigalleau, Guy Touati, Luis Aldamiz-Echevarria, Pascal Cathebras, Didier Eyer, Dominique Brunet, Léna Damaj, Dries Dobbelaere, Claire Gay, Sylvie Hiéronimus, Virginie Levrat, François Maillot

**Affiliations:** 1 Hôpital Necker-Enfants Malades, Paris, France; 2 Sanofi Genzyme, 500 Kendall St, Cambridge, MA 02140 USA; 3 Hôpital Robert Debré, Paris, France; 4 Hôpital Femme Mère Enfant, Bron, France; 5 Hôpital Pitié-Salpêtrière, Paris, France; 6 Hospital St Joan de Déu, Barcelona, Spain; 7 Hospital Universitario Ramon y Cajal, Madrid, Spain; 8 Center of Reference for Inborn Metabolic Disease, CHU La Timone, Marseille, France; 9 Hospital Clínico Universitario, Santiago de Compostela-La Coruña, Spain; 10 Hospital Universitario y Politécnico La Fe, Valencia, Spain; 11 Hospital Universitario Materno-Infantil, Universidad de Las Palmas de Gran Canaria, CIBER OBN, Las Palmas, Spain; 12 Hôpital Haut Leveque, Pessac, France; 13 Hôpital des Enfants, Toulouse, France; 14 Hospital Universitario Cruces-Osakidetza, Barakaldo-Vizcaya, Spain; 15 CHU Hôpital Nord, Saint-Etienne, France; 16 CHU Hautepierre, Strasbourg, France; 17 CHU La Timone, Marseille, France; 18 CHU Hôpital Sud, Rennes, France; 19 Medical Reference Center for Inherited Metabolic Diseases, Jeanne de Flandre University Hospital and RADEME Research Team for Rare Metabolic and Developmental Diseases, Lille, France; 20 Hôpital l’Archet, CHU de Nice, France; 21 Centre Hospitalier Annecy Genevois, Pringy, France; 22 CHRU de Tours, Service de Médecine Interne, Université François Rabelais, Tours, France

**Keywords:** Betaine anhydrous, Efficacy, Homocystinuria, RoCH registry, Safety

## Abstract

**Background:**

The Registry of Adult and Paediatric Patients Treated with Cystadane® – Homocystinuria (RoCH) is a non-interventional, observational, multi-centre, post-authorization safety study that aimed to identify safety of betaine anhydrous (Cystadane®) in the treatment of patients with inborn errors of homocysteine metabolism (homocystinuria) in order to minimise the treatment associated risks and establish better knowledge on its clinical use. The registry included patients of all ages with homocystinuria who were treated with betaine anhydrous in conjunction with other therapies. Clinical data were collected retrospectively from 2007 to 2013, then prospectively up to February 2014. All adverse events (AEs) reported during the study were recorded. The clinical and biological status of patients was monitored at least once a year.

**Results:**

A total of 125 patients with homocystinuria (adults [> 18 years]: 50; paediatric [≤18 years]: 75) were enrolled at 29 centres in France and Spain. Patients were treated with betaine anhydrous for a mean duration of 7.4 ± 4.3 years. The median total daily dose of betaine anhydrous at the first and last study visits was 6 g/day for cystathionine β-synthase (CBS)-deficient vitamin B6 responders and 9 g/day for methylenetetrahydrofolate reductase-deficient patients, while the median daily dose increased in CBS-deficient B6 non-responders (from 6 to 9 g/day) and cobalamin metabolism-defective patients (from 3 to 6 g/day) between the first and last visits. Treatment caused a mean overall reduction of 29% in plasma homocysteine levels in the study population. A total of 277 AEs were reported during the study, of which two non-serious AEs (bad taste and headache) and one serious AE (interstitial lung disease) were considered to be drug related. Overall, betaine anhydrous was well tolerated with no major safety concerns.

**Conclusions:**

Data from the RoCH registry provided real-world evidence on the clinical safety and efficacy of betaine anhydrous in the management of homocystinuria in paediatric and adult patients.

**Electronic supplementary material:**

The online version of this article (10.1186/s13023-019-1036-2) contains supplementary material, which is available to authorized users.

## Background

Homocystinuria is an autosomal recessive disorder of homocysteine metabolism leading to increased plasma, urine and tissue accumulation of homocysteine and its metabolites [1, 2]. Under normal conditions, the amino acid methionine is transmethylated to homocysteine, a sulphur amino acid not used for protein synthesis [1]. Homocysteine is either irreversibly degraded to cysteine by the vitamin B6-dependent enzymes cystathionine β-synthase (CBS) and cystathionine-γ-lyase or remethylated back to methionine by methionine synthase (MS) [3]. Inherited disorders of homocysteine metabolism therefore include disorders of the trans-sulphuration pathway with CBS deficiency, known as classical homocystinuria, and remethylation disorders [1, 2]. The clinical manifestations of classical homocystinuria include skeletal abnormalities, osteoporosis, ectopia lentis and/or severe myopia, cognitive impairment, developmental delay/intellectual disability, seizures, psychiatric disorders, atherosclerosis and thromboembolism [4]. If untreated, homocystinuria is a serious, life-threatening disease [3, 5].

The two phenotypic variants of CBS deficiency are B6-responsive and B6-non-responsive homocystinuria, where mutations in the CBS gene cause a varying response to vitamin B6 [6–10]. CBS deficiency results in increased plasma and tissue homocysteine and methionine levels [11]. Remethylation disorders include 5,10-methylenetetrahydrofolate reductase (MTHFR) deficiency and errors of cobalamin (Cbl, vitamin B12) metabolism [2]. MTHFR converts 5,10-methyltetrahydrofolate (5,10-MTHF) into 5-MTHF, the most biologically active form of vitamin B9, which acts as a methyl donor and functions together with vitamin B12 in the conversion of homocysteine to methionine [12]. Defects in the steps of Cbl metabolism include abnormalities in dietary intake, intestinal absorption, blood transport of Cbl by transcobalamin (TC), cellular uptake and intracellular metabolism (cblF, cblJ, cblC, cblD, cblE and cblG defects) [13–15].

Betaine anhydrous (Cystadane®, Orphan Europe) is a methylating agent approved for the treatment of homocystinuria involving CBS, MTHFR or cbl defects [16, 17]. Betaine anhydrous acts as a methyl donor for remethylation of homocysteine to methionine, increasing the plasma and tissue concentrations of methionine while reducing homocysteine levels [16]. The efficacy of betaine anhydrous in the treatment of homocystinuria has been well established [3, 7, 8, 18–20]. The Registry of Adult and Paediatric Patients Treated with Cystadane® - Homocystinuria (RoCH) was a post-authorization safety study (PASS) conducted in Europe to identify adverse events (AEs) associated with the use of betaine anhydrous in clinical practice, with an aim of minimizing potential risks with treatment and establishing better clinical knowledge about its use in patients with homocystinuria. The results of the RoCH registry study are presented here.

## Methods

### Registry design

This open-label, non-comparative and non-interventional study included patients of all ages with homocystinuria who were treated with betaine anhydrous in conjunction with other therapies and had available clinical data from at least two study visits (before and after betaine anhydrous treatment). Patients who were not treated with betaine anhydrous were excluded, along with those who did not wish to participate. Patients provided verbal approval prior to entry into the registry.

Clinical data of all patients included in the study were assessed by the treating physician participating in the registry. At the inclusion visit and at each follow-up visit, all ocular, skeletal, cardiovascular, neurological, psychiatric and other miscellaneous abnormalities (Additional file 1: Table S1) were specified on the clinical observation form and assessed for each patient, along with brain and spinal cord nuclear magnetic resonance imaging. Plasma levels of total homocysteine, methionine, vitamin B12, free homocysteine and folates, and urinary levels of methylmalonic acid were also recorded at each visit. Severe homocystinuria was defined as total plasma homocysteine level of > 50 μmol/L.

Data on betaine anhydrous treatment (start and stop dates, dose and frequency), methionine-restricted diet and other relevant concomitant medications were also collected at each visit. Safety data were recorded on specific safety reporting forms and submitted to the pharmacovigilance department of Orphan Europe, where it was entered in the global safety database and evaluated according to the European guidelines [1, 2] and legislation in pharmacovigilance [21].

### Statistical analysis

Descriptive statistics were used. For quantitative variables, the sample size, mean, median, standard deviation (SD), standard error of the mean, coefficient of variation, minimum and maximum values, and quartiles were provided. For qualitative variables, sample size and absolute and relative frequencies per class were provided.

## Results

All data included in the study were collected retrospectively from 2007 to 2013, after which it was prospectively collected up to 28 February 2014. The clinical and biological status of each patient was monitored at least once a year for most patients. A total of 623 visit forms were collected.

### Patients

A total of 125 patients (56.8% male; mean age 16.80 ± 14.4 years) were enrolled from 29 European centres (22 in France and 7 in Spain) during the study period (Table 1). The most common diagnosis of type of homocystinuria at baseline was B6 non-responsive CBS deficiency (*n* = 49, 39.2%), followed by Cbl metabolism defects (*n* = 45, 36.0%), MTHFR deficiency (*n* = 21, 16.8%), and B6 responsive CBS deficiency (*n* = 9, 7.2%; Table 1). The majority of patients in all diagnostic categories were male, except for an approximately 1:1 male:female ratio observed in patients with Cbl metabolism defects and MTHFR deficiency (Table 1).

**Table 1:** Baseline characteristics of patients included in the study

	CBS deficiency vitamin B6 responder (*N* = 9)	CBS deficiency vitamin B6 non-responder (*N* = 49)	MTHFR deficiency (*N* = 21)	Cbl metabolism defects (*N* = 45)	Total (*N* = 125)^a^
Age, years					
Mean ± SD	34.41 ± 14.4	20.42 ± 12.3	18.14 ± 14.5	8.04 ± 10.3	16.80 ± 14.4
Median (min, max)	39 (17.6, 56.9)	18 (0.1, 55.5)	20 (0.0, 56.5)	6 (0.0, 44.33)	14 (0.0, 56.9)
Sex, n (%)					
Female	2 (22.2)	18 (36.7)	11 (52.4)	22 (48.9)	54 (43.2)
Male	7 (77.8)	31 (63.3)	10 (47.6)	23 (51.1)	71 (56.8)
Age at onset of first symptoms, years					
n	9	41	20	44	114
Mean ± SD	26.01 ± 14.7	5.79 ± 6.3	8.69 ± 12.8	3.02 ± 8.3	6.83 ± 10.9
Median (min, max)	31 (0.8, 48.0)	4 (0.0, 28.7)	2 (0.0, 50.2)	0 (0.0, 41.2)	2 (0.0, 50.2)
Age at diagnosis, years					
n	9	49	20	45	124
Mean ± SD	28.10 ± 17.7	7.27 ± 6.2	10.32 ± 14.5	3.45 ± 9.2	8.21 ± 12.2
Median (min, max)	31 (0.8, 56.6)	6 (0.0, 28.7)	5 (0.0, 56.5)	0 (0.0, 42.2)	4 (0.0, 56.6)
Delay between onset of symptoms and diagnosis, years					
n	9	41	20	44	114
Mean ± SD	2.10 ± 5.6	2.15 ± 3.2	1.62 ± 2.3	0.50 ± 1.5	1.42 ± 2.9
Median (min, max)	0 (0.0, 17.0)	1 (0.0,15.0)	0 (0.0, 6.3)	0 (0.0, 7.6)	0 (0.0, 17.0)
Total plasma homocysteine, μmol/L					
n	9	45	18	41	114
Mean ± SD	118.29 ± 80.63	115 ± 72.86	146.2 ± 78.35	90.54 ± 56.6	111.29 ± 70.31
Median (min, max)	96 (15.0, 249.9)	98 (12.7, 277)	122.3 (64.5, 389)	71.5 (21.2, 228)	95.45 (12.7, 389)

*Cbl* cobalamin, *CBS* cystathionine β-synthase, *MTHFR* 5, 10-methylenetetrahydrofolate reductase, *SD* standard deviation

^a^ Includes patient who was CBS-deficient vitamin B6 non responder and MTHFR-deficient

Of the 45 patients with Cbl metabolism defects, 42 patients had known Cbl deficiency type at the first visit: 88.1% were cblC-defective (*n* = 37), 9.5% were cblE/G-defective (n = 4), and 2.4% (*n* = 1) had TC deficiency. A 46-year-old female patient was reported with a double diagnosis (CBS-deficient vitamin B6 non-responder and MTHFR-deficient).

The median age of patients was 39 years for CBS-deficient B6 responders, 18 years for CBS-deficient B6 non-responders and 20 years for MTHFR-deficient patients (Table 1). Cbl metabolism-defective patients were the youngest, with a median age of 6 years, and were diagnosed with homocystinuria the earliest, at a mean age of 3.5 ± 9.2 years (median in the first year of life) and 3.02 ± 8.3 years after the first onset of symptoms (Table 1). CBS-deficient B6 responders were diagnosed at a much later age compared with the other diagnostic categories, with a mean age of 28.1 ± 17.7 years (median 31 years) (Table 1).

### Treatment

All patients included in the study received betaine anhydrous treatment for a mean duration of 7.4 ± 4.3 years (median 7 years [0.0–22.8]). Prior to being treated with betaine anhydrous, 26 patients had received treatment with other formulations of betaine, all of which were discontinued immediately upon prescription of betaine anhydrous.

The median total daily dose of betaine anhydrous was 6 g/day for CBS-deficient B6 responders recorded at both the first and last visits. MTHFR-deficient patients were treated with a higher dose, with a median value of 9 g/day at first and last visits. The median total daily dose between the first and last visits increased from 6 to 9 g/day in CBS-deficient vitamin B6 non-responders, and from 3 to 6 g/day in Cbl metabolism-defective patients.

**Fig. 1:**
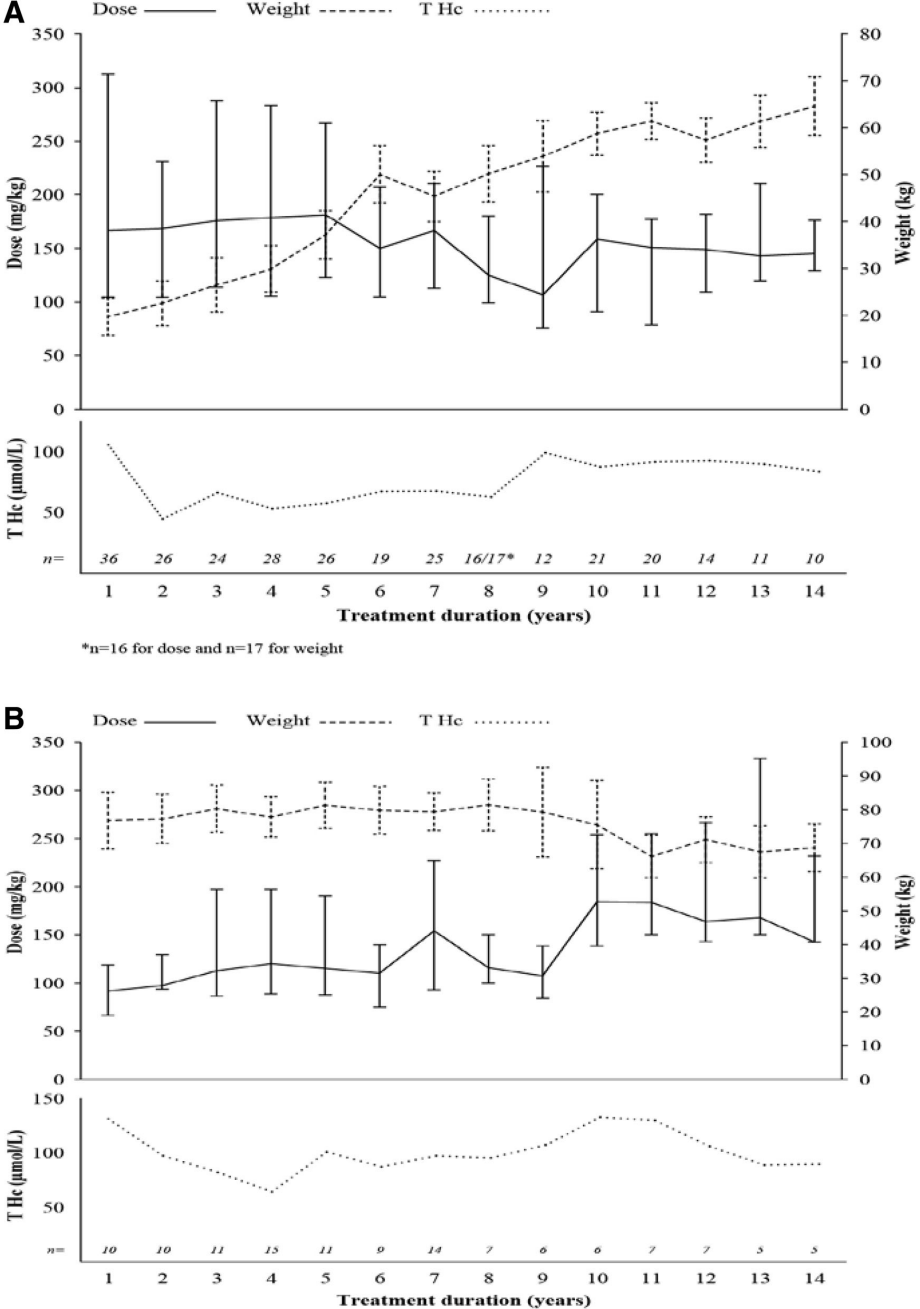
Betaine anhydrous total daily dose and patient weight in (**a**) paediatric and (**b**) adult patients. Values correspond to mean ± standard error for weight (kg) and mean for total homocysteine (T Hc, μmol/L) during the treatment duration. Median, Q1 and Q3 values are presented for dose (mg/kg)

To exclude the impact of increasing body weight on the prescribed dose of betaine anhydrous in paediatric patients, the median total daily dose was recalculated as mg/kg/day and varied between 108 and 167 mg/kg/day in the overall population. The median total daily dose of betaine anhydrous varied from 107 to 181 mg/kg/day in the paediatric population (*n* = 75). Paediatric patients received a higher dose of betaine anhydrous during the first year of treatment compared with later years. Specifically, paediatric patients with MTHFR and Cbl metabolism defects received higher doses of betaine anhydrous compared with the other two diagnostic groups (data not shown). Mean levels of total plasma homocysteine in the paediatric subgroup varied between 52.9 and 105.7 μmol/L (Fig. 1a).

The median total daily dose of betaine anhydrous in adult population (*n* = 50) varied between 92 and 184 mg/kg/day, and the mean levels of total homocysteine were within a range of 63.8–131.0 μmol/L. After 9 years of treatment, adult patients received higher doses of betaine anhydrous (Fig. 1b), which could probably be due to decrease in body weight of these patients, as the dose of betaine anhydrous was not adjusted according to change in weight in these patients during the treatment period.

All CBS-deficient B6 responders were treated with vitamin B6 and folic acid in addition to betaine anhydrous, and 88.9% of these patients were also treated with vitamin B12. CBS-deficient B6 non-responders were mainly supplemented with vitamin B6 (91.8%), folic acid (83.7%) and oral vitamin B12 (61.2%), and 87.8% followed a methionine-restricted diet. MTHFR-deficient patients were mainly supplemented with folinic acid (76.2%) and oral vitamin B12 (57.1%), and Cbl metabolism-defective patients were mostly supplemented with intramuscular vitamin B12 (93.3%), carnitine (80.0%) and folinic acid (71.1%; Table 2).

**Table 2:** Concomitant medications used during the study

Concomitant medications	CBS deficiency, %		MTHFR deficiency, %	Cbl metabolism defects, %
	Vit B6 responders	Vit B6 non-responders		
Carnitine	11.1	12.24	33.33	80
Oral Vit B12	88.69	61.22	57.14	35.56
SC Vit B12	–	–	4.76	11.11
IM Vit B12	–	24.49	38.10	93.33
Folinic acid	–	12.24	76.19	71.11
Vit B6	100	91.84	33.33	26.67
Folic acid	100	83.67	38.10	46.67
L-methionine	–	4.08	28.57	31.11
Other forms of betaine	–	22.45	23.81	22.22
Methionine restricted diet	–	87.76	28.57	35.56
MTHF	–	–	19.05	–
Riboflavine	–	–	28.57	–

*Cbl* cobalamin, *IM* intramuscular, *MTHF* methylenetetrahydrofolate, *MTHFR* 5,10 methylenetetrahydrofolate reductase; SC, subcutaneous, *Vit* vitamin

### Disease characteristics

A broad range of clinical presentations associated with homocystinuria were observed during the study period (Additional file 2: Figure S1). CBS-deficient vitamin B6 non-responders mainly reported ocular (74.0%), skeletal (72.0%), neurologic (66.0%) and cardiovascular (46.0%) symptoms. Skeletal and cardiovascular issues were reported by 77.8% of CBS-deficient B6 responders, followed by ocular (66.7%) and neurologic (55.6%) abnormalities. The majority of Cbl metabolism-defective patients (80.0%) reported neurologic disorders, followed by ocular and hematologic disorders in 66.7 and 64.4% of patients, respectively. All MTHFR-deficient patients had neurologic impairment, over half (52.4%) had psychiatric disturbances, and skeletal disorders were reported in 38.1% of these patients (Additional file 2: Figure S1).

### Biochemical analysis

**Fig. 2:**
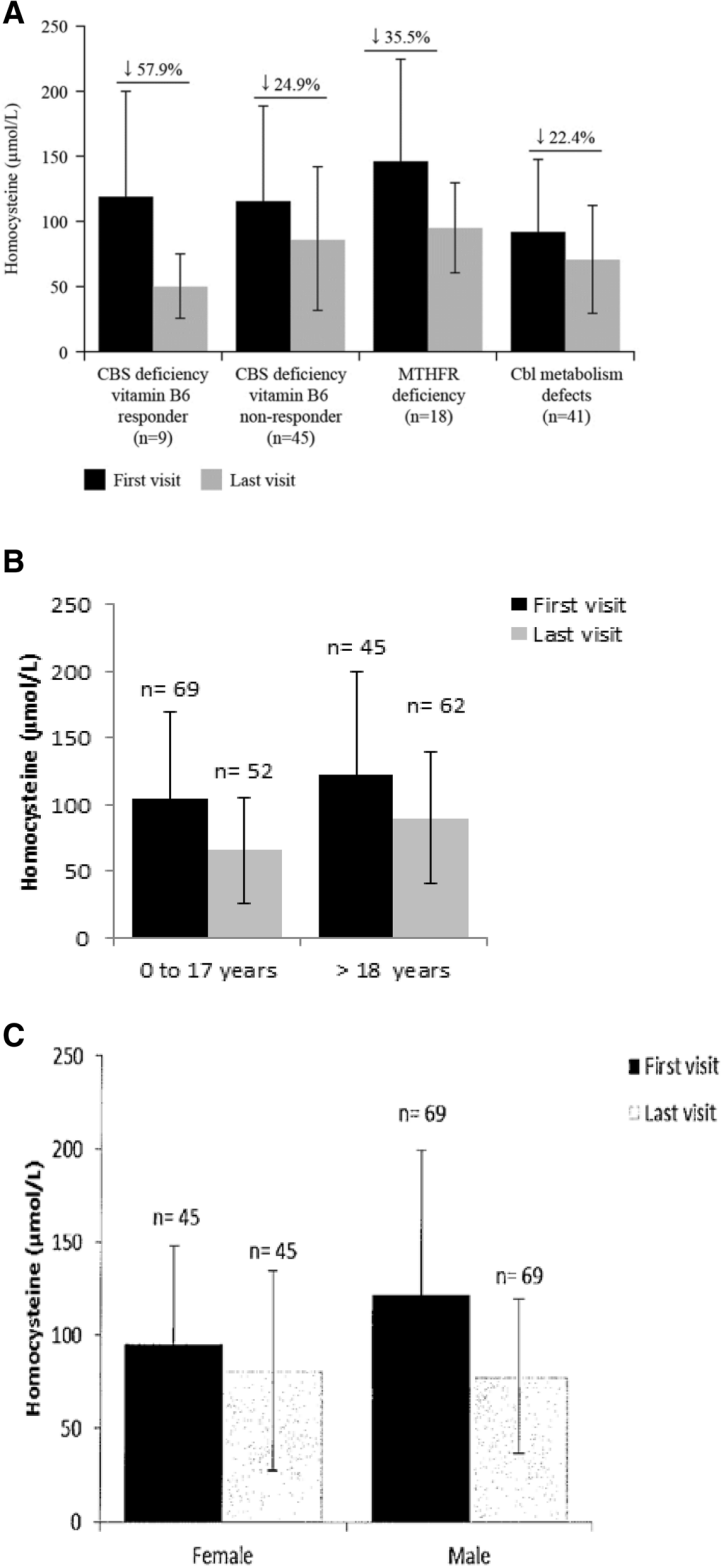
Mean plasma homocysteine levels stratified by (**a**) diagnostic groups, (**b**) age, and (**c**) gender

Laboratory values were available for patients who had data for at least two visits to the registry. Mean total plasma homocysteine levels at the last visit were decreased compared with the first visit in all diagnostic groups (79.07 ± 46.51 μmol/L versus 111.29 ± 70.31 μmol/L), with a mean overall reduction of 29.0%. Among the subgroups, mean decrease was highest in the CBS-deficient B6 responders (57.9%; Fig. 2a). Total homocysteine levels decreased after treatment with betaine anhydrous regardless of age group and gender (Fig. 2b and c).

In patients with severe homocystinuria, total plasma homocysteine levels decreased from 129.3 μmol/L (*n* = 93) at the first visit to 96.3 μmol/L (*n* = 82) at the last visit, at which the mean total homocysteine levels were < 100 μmol/L.

**Fig. 3:**
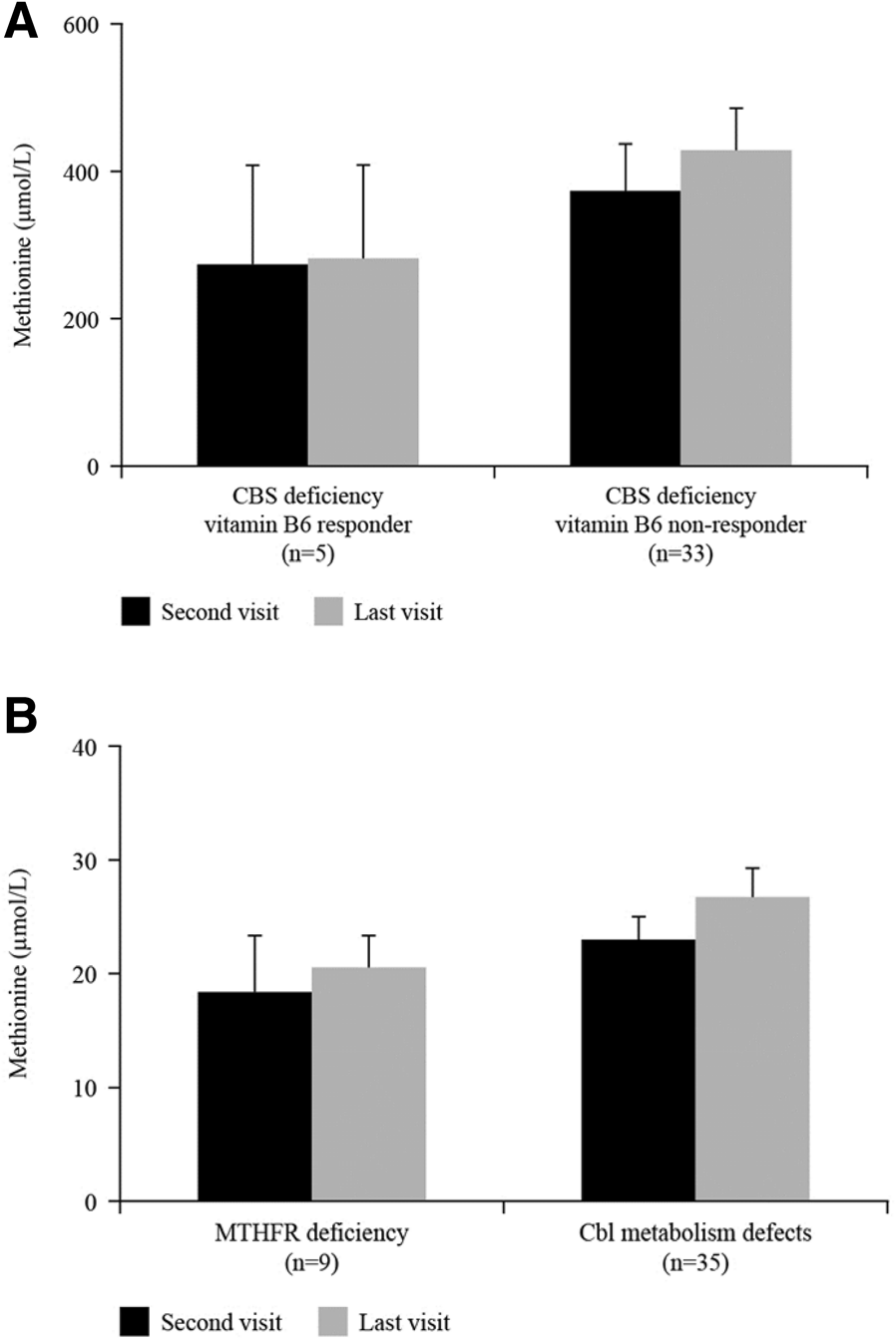
Mean methionine levels in (**a**) CBS-deficient and (**b**) MTHFR- and Cbl-deficient patients during the study. Cbl, cobalamin; CBS, cystathionine β-synthase; MTHFR, 5, 10-methylenetetrahydrofolate reductase

As some patients started treatment with betaine anhydrous only after the first visit, methionine levels were analysed at the second visit to ensure that all patients were on treatment. Mean methionine levels were increased in both sub-types of CBS deficiencies (273.2 μmol/L in B6 responders and 374.9 μmol/L in B6 non-responders) compared with the other two diagnostic groups (Fig. 3a and b). A slight increase was observed in B6 non-responders (428.7 μmol/L) at the last visit, but values remained well below the safety threshold of 1000 μmol/L. As expected, methionine levels were low in MTHFR- and Cbl metabolism-defective patients (18.6 and 23.4 μmol/L, respectively [normal values 16–30 μmol/L]); a slight increase was observed at the last visit (Fig. 3b).

### Evolution of clinical symptoms

Overall, clinical symptoms of disorders associated with homocystinuria improved in 28.7% of patients, stabilised in 69.7% of patients and worsened in 1.6% of patients at the end of the study period. Among patients with clinical improvement of symptoms, an improvement of 33.0, 31.5, 26.6 and 13.6% was observed in Cbl metabolism-defective, MTHFR-deficient, CBS-deficient B6 non-responders, and CBS-deficient B6 responders, respectively. Clinical improvement of symptoms was determined by the treating physician at each visit as no change, improvement or worsening compared with previous visits.

### Safety

A total of 277 AEs were reported by System Organ Class (SOC) during the study period, of which 109 were serious. The most frequently reported AEs were: injury, poisoning and procedural complications (16.1%), infections and infestations (15.5%), gastrointestinal disorders (9.75%), nervous system disorders (9.0%), musculoskeletal and connective tissue disorders (7.2%), and respiratory, thoracic and mediastinal disorders (7.2%). Only two non-serious AEs (bad taste and headache) and one serious AE (interstitial lung disease) were assessed as being possibly related to betaine anhydrous.

Interstitial lung disease was reported as a serious AE in a 2-year-old cblC-defective male patient treated with betaine anhydrous (2 g/day) who experienced respiratory distress related to pulmonary hypertension and interstitial lung disease leading to death.

Two other deaths occurred during the registry. A 9-year-old CBS-deficient B6 non-responder male patient treated with 6 g/day of betaine anhydrous (initiated after the start of the event) experienced brain vein thrombosis which led to brain oedema and intracranial hypertension. The patient was diagnosed 2 days prior to the start of treatment and methionine levels were 433 μmol/L a day prior to death. A 15-month old MTHFR-deficient female patient treated with 3 g/day betaine anhydrous died of respiratory distress. These deaths were not considered as related to treatment.

There were eight cases of betaine anhydrous exposure during pregnancy: five resulted in the delivery of healthy babies and three resulted in spontaneous abortion, none of which were presumed to be related to betaine anhydrous.

## Discussion

The results of this registry provide real-world information on the use of betaine anhydrous in the management of homocystinuria in clinical practice. Betaine anhydrous was associated with decreased total plasma homocysteine, increased methionine, and improvement/stabilisation of the clinical symptoms of different disorders associated with homocystinuria in the treated population. Overall, betaine anhydrous was well tolerated, with interstitial lung disease being the only serious AE reported in one patient that was considered likely to be associated with treatment.

Plasma homocysteine levels generally determine the severity of homocystinuria [22]. One of the aims of betaine anhydrous treatment is to keep plasma total homocysteine levels as low as possible [23]. As per the current guidelines, additional treatment such as betaine and/or dietary modifications should be considered in CBS-deficient B6 responders in whom total homocysteine levels remain above 50 μmol/L [1]. These guidelines also highlight that patients are unlikely to develop complications if plasma homocysteine levels are maintained below 120 μmol/L, with the recommendation to maintain levels below 100 μmol/L to allow for natural homocysteine fluctuations [1]. In this RoCH registry study, betaine anhydrous treatment decreased total plasma homocysteine levels below the recommended 100 μmol/L in the total study population, regardless of the nature of the deficiency (79.07 ± 46.51 μmol/L vs 111.29 ± 70.31 μmol/L at baseline), and the clinical symptoms of disorders associated with homocystinuria either improved or stabilised in the majority of patients during the study period.

In the present study population, betaine anhydrous was well tolerated, with manageable AEs. Bad taste and headache were the main non-serious AEs associated with treatment, and interstitial lung disease was the only serious AE leading to death. Interstitial lung disease occurred after 1 year of betaine anhydrous treatment and was considered to be treatment-related as no other reason for its occurrence could be determined. However, since only one case of interstitial lung disease was reported during the study period, it could be idiopathic, but has been included in the risk management plan as a potential important risk with betaine anhydrous treatment due to its seriousness.

The main safety issue with the use of betaine anhydrous is the possibility of elevated methionine levels formed by homocysteine remethylation, which may lead to cerebral oedema [3, 24]. Elevated methionine levels (> 1000 μmol/L) are a major concern in CBS-deficient patients, and cerebral oedema has previously been reported in these patients when treated with betaine anhydrous [6, 25–27]. Since the majority of patients in the present study followed a methionine-restricted diet along with treatment, methionine levels of CBS-deficient patients remained well below the critical threshold of 1000 μmol/L, which is an important safety-monitoring parameter for the treatment of this diagnostic group. The slight increase in methionine levels in MTHFR- and Cbl metabolism-defective patients may reflect the efficacy of treatment in these subgroups.

CBS deficiency is generally characterised by developmental delay, intellectual disability, ocular complications, skeletal abnormalities and thromboembolism, with the expression of these clinical signs being extremely varied [3, 10, 28]. During the present study, clinical presentations of homocystinuria were wide-ranging and varied depending on the specific genetic defect. The majority of B6 non-responders presented ocular and skeletal disorders, while CBS-deficient B6 responders presented skeletal and cardiovascular disorders, followed by ocular complications. Although all these deficiencies resulted from increased homocysteine levels, patients in each diagnostic subgroup presented different symptoms and required different disease management.

The median age of patients varied across diagnostic groups, Cbl metabolism-defective patients being the youngest, and all diagnostic groups except the CBS-deficient B6 responders were diagnosed in childhood. Furthermore, the delay between the onset of first symptoms and diagnosis among Cbl-deficient patients was the shortest compared with other deficiencies. In Cbl metabolism-defective patients, clinical features included acute neurologic deterioration, developmental delay, lethargy, hypotonia and feeding problems in neonatal (< 1 month old) and early onset patients (< 1 year of life). The severity of initial manifestations in this subgroup may explain the early diagnosis as well as the short delay between the onset of symptoms and diagnosis.

Currently, newborn screening programs allow the diagnosis of CBS deficiency, detected by elevated methionine and homocysteine levels [29, 30]. However, this approach only allows the detection of the B6 non-responsive form, and rarely detects B6-responsive CBS-deficient newborn babies as increase in methionine is not obvious in these infants [30]. Very often the diagnosis of B6 responders is still made only after the development of complications that are mainly irreversible [4, 6]. This could explain the higher median age of diagnosis, and the delay between the onset of symptoms and diagnosis of homocystinuria in CBS-deficient B6 responders in the present study.

The clinical signs of remethylation defects are known to be mainly neurologic [31–33]. The main abnormalities observed in remethylation defective patients at baseline in this registry were indeed neurologic, with 80% of Cbl metabolism-defective and 100% of MTHFR-deficient patients displaying neurologic abnormalities. If untreated, these patients may develop acute or rapidly progressive neurologic deterioration, sometimes leading to death [4]. In the present study, betaine anhydrous treatment improved or stabilised the overall clinical symptoms in the majority of patients, indicating that betaine anhydrous effectively controls the symptoms of homocystinuria when used in conjunction with other relevant medications.

The recommended total daily dose of betaine anhydrous in adult and paediatric patients > 10 years of age is 6 g/day [1]. During the present study, CBS-deficient B6 responders were treated with a median dose of 6 g/day, whereas MTHFR-deficient patients were treated with a higher dose of betaine anhydrous (9 g/day), and the median total daily dose for CBS-deficient B6 non-responders and Cbl-deficient patients increased between the first and last visits. These data are consistent with literature reports, where betaine is administered at a higher dose of 5–20 g/day in adults and 150–250 mg/kg/day in children [4]. Since the majority of patients included in the study were ≤ 18 years of age (*n* = 75), in whom the adult dose of 6 g/day was to be administered from the age of 10 years, many investigators may have continued to adjust treatment to weight in paediatric patients aged > 10 years, which would explain the increase of total daily dose of betaine anhydrous recorded at the last visit.

The main limitation of the present study is that data was not statistically analysed to determine the significance of changes in different parameters observed during the study period. Also, the study was designed mainly to assess safety of betaine anhydrous and enrolled a highly heterogeneous patient population, which did not allow for statistical confirmation of changes in plasma homocysteine or methionine levels. Furthermore, data were collected retrospectively during most of the study period, which may have introduced bias.

## Conclusion

Overall, the RoCH registry provides a better understanding of the clinical management of homocystinuria in different diagnostic subgroups. The results from this registry show that betaine anhydrous is well tolerated in patients with homocystinuria and highlights the importance of maintaining methionine levels below the safety threshold of 1000 μmol/L in patients with CBS defects as these patients have a higher risk of AEs due to hypermethioninemia. Further data characterizing the real-world use of betaine anhydrous in patients with homocystinuria are being collected through partnership with E-HOD (European network and registry for homocystinurias and methylation defects).

## Additional files

Additional file 1: **Table S1.** Abnormalities recorded at inclusion visit and each follow-up visit among patients included in the study. (TIFF 4325 kb)

Additional file 2: **Figure S2.** Distribution of presenting symptoms of homocystinuria during the study. Cbl, cobalamin; CBS, cystathionine β-synthase; MRI, magnetic resonance imaging; MTHFR, 5, 10-methylenetetrahydrofolate reductase. (DOCX 15 kb)

## Abbreviations

AE: Adverse events
Cbl: Cobalamin
CBS: Cystathionine β-synthase
MS: Methionine synthase
MTHF: Methyltetrahydrofolate
MTHFR: 5,10 methylenetetrahydrofolate reductase
PASS: Post-authorization safety study
RoCH: Registry of Adult and Paediatric Patients Treated with Cystadane® - Homocystinuria
SD: Standard deviation
SOC: System organ class
TC: Transcobalamin
