# Supplementary material for: Betaine anhydrous in homocystinuria: results from the RoCH registry
Source: Orphanet J Rare Dis. 2019 Mar 14;14:66. doi: 10.1186/s13023-019-1036-2 (PMC6419445; doi:10.1186/s13023-019-1036-2)
Supplement: Supplementary file 2 — Figure S2. Distribution of presenting symptoms of homocystinuria during the study. Cbl, cobalamin; CBS, cystathionine β-synthase; MRI, magnetic resonance imaging; MTHFR, 5, 10-methylenetetrahydrofolate reductase. (DOCX 15 kb) [file 13023_2019_1036_MOESM2_ESM.docx]

**Supplementary Material**

**Supplementary Table 1.** Abnormalities recorded at inclusion visit and each follow-up visit among patients included in the study.

| **Body system** | **Specific abnormalities** |
| --- | --- |
| Ocular | Ectopia lentis  Myopia  Iridodonesis  Optic atrophy  Retinal detachment  Corneal abnormalities  Glaucoma  Retinal degradation  Cataract |
| Skeletal | Osteoporosis  Biconcave vertebrae  Scoliosis  Increased length of long bones  Irregular widened metaphyses  Metaphyseal spicules  Abnormal size and shape of epiphysis  Growth arrest lines  Pes cavus  High-arched palate  Genu valgum  Kyphosis  Short fourth metacarpal |
| Cardiovascular | Venous thrombosis  Peripheral arterial thrombosis  Pulmonary embolism  Doppler abnormalities  Stroke  Myocardial infarction  Electrocardiogram abnormality  Malar flush  Livedo reticularis |
| Neurological | Cognitive decline  Extrapyramidal signs  Pyramidal syndrome  Ataxia  Dystonia  Hypotonia  Hemiparaparesis  Spastic paraparesis  Myopathy  Myelopathy  Coma  Seizures  Electroencephalogram abnormality |
| Psychiatric | Mental retardation  Depression  Obsessive compulsive disorder  Personality disorder  Behaviour disorder  Hallucinosis  Delusion  Catatonia |
| Miscellaneous | Fatty changes in liver  Inguinal hernia  Endocrine abnormalities  Reduced clotting factors  Megaloblastic anaemia  Respiratory failure  Macrocytosis  Proteinuria  Haemolytic uremic syndrome  Glomerulonephritis |
